# Letter from Dr. W. H. Morgan, D. D. S.

**Published:** 1874-02

**Authors:** W. H. Morgan


*Letter from Dr. W. H. Morgan.*

NASHVILLE, January 2nd, 1874.

MR. EDITOR:—In the December No. of *Missouri Dental Journal*, I find this paragraph: "In an obituary notice of the late Dr. Amos Wescott, the author states that the organization of the Baltimore College of Dental Surgery was due to the efforts of Dr. Wescott. While we admit that Dr. Wescott was a very efficient member of the Faculty of this institution in its early days, we think that great injustice has been done to others in making a statement of this nature which is so wide of the truth;" and further on we have the following: "Dr. Amos Wescott did not become a member of the Faculty of this institution until 1846, and resigned in 1849, as the records of the College now in the possession of the writer will show." This leaves us to infer that he was an efficient member for two years or two sessions at least; such is not the fact, as reference will show that the undersigned attended that college during the sessions of '47 and '48, and here states that Dr. Wescott was not present, and therefore did not deliver a lecture in the College during that term, closing March 1st, 1843. This statement can be verified by Dr. A. A. Blandy and Charles Ballard, of London, Dr. John McCalla, of Lancaster, Pa., and Dr. T. J. Jones, of Ga.

Very respectfully,

W. H. MORGAN.

[ We can explain by stating that Dr. Wescott held the position of Demonstrator of Operative Dentistry for one session prior to 1846, and was then elected Professor of Theory and Practice of Dental Surgery, and although he was not present during the session of 1847–48, yet his resignation was not acted upon until the close of this latter session, hence his name remained upon the books as filling the Chair referred to.]—ED.

